# Intramyocardial Dissection following Postinfarction Ventricular Wall Rupture Contained by Surrounding Postoperative Adhesions

**DOI:** 10.1155/2015/584795

**Published:** 2015-03-19

**Authors:** Abdulkadir Ercan, Orcun Gurbuz, Gencehan Kumtepe, Hakan Ozkan, Ilker Hasan Karal, Yusuf Velioglu, Serdar Ener

**Affiliations:** ^1^Department of Cardiovascular Surgery, Balıkesir University Medical Faculty, Bigadiç, 10145 Balıkesir, Turkey; ^2^Department of Cardiology, Bahçeşehir University Faculty of Medicine, Istanbul, Turkey; ^3^Department of Cardiovascular Surgery, Samsun Hospital for Education and Research, İlkadım, 55090 Samsun, Turkey; ^4^Department of Cardiovascular Surgery, Medical Park Uşak Hospitals, Uşak, Turkey; ^5^Department of Cardiovascular Surgery, Acıbadem Bursa Hospital, Bursa, Turkey

## Abstract

*Introduction*. Dissection of the myocardium is a rare form of cardiac rupture, caused by a hemorrhagic dissection among the spiral myocardial fibers, its diagnosis is rarely established before the operation or death, and extremely few cases have been reported in the literature and none of these cases seem to have a history of previous cardiac surgery which makes our report unique. *Case Presentation*. A 61-year-old female patient was admitted into the emergency room with complaints of progressive chest pain for 2 days. She had a history of second time prosthetic aortic valve replacement and was under anticoagulation therapy. She was diagnosed with an acute inferoposterior myocardial infarction and underwent emergency coronary angiography revealing spontaneous recanalization of the right coronary artery. During the follow-up, she developed cardiogenic shock and a new occurring systolic ejection murmur. Transthoracic echocardiography showed a left ventricular free wall rupture; then, she was taken in for emergency surgery. During the operation, a rupture zone and a wide intramyocardial dissecting area were detected. Intraventricular patch repair technic with autologous pericardial patch was used to exclude the ruptured area. Following the warming period, despite adequate hemostasis, hemorrhage around suture lines progressively increased, leading to the patient's death. *Conclusion*. Pericardial adhesions might contain left ventricular rupture leading to intramyocardial dissection.

## 1. Introduction

Intramyocardial dissection is a rarely reported condition, caused by a dissection among the spiral myocardial fibers due to ischemia. Its diagnosis is often challenging and in most cases it is observed postmortem. We present the case report of a 61-year-old woman who suffered a spontaneous recanalized acute inferoposterior myocardial infarction causing intramyocardial dissection and left ventricular free wall rupture contained by surrounding adhesions due to previous aortic surgery, which makes our report unique.

## 2. Case Presentation

**Figure 1:**
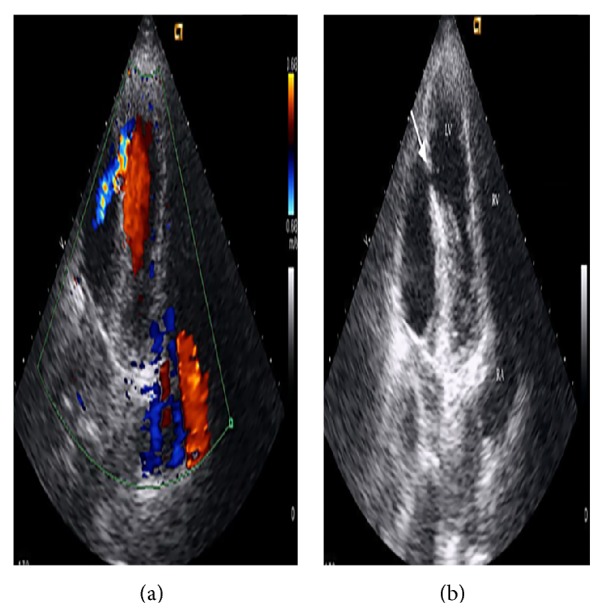
Modified apical four-chambered view in transthoracic echocardiography showing the defect (b) (marked by white arrow) and color jet (a) through it. LV: left ventricle, RV: right ventricle, RA: right atrium, and IMD: intramyocardial dissection area.

**Figure 2:**
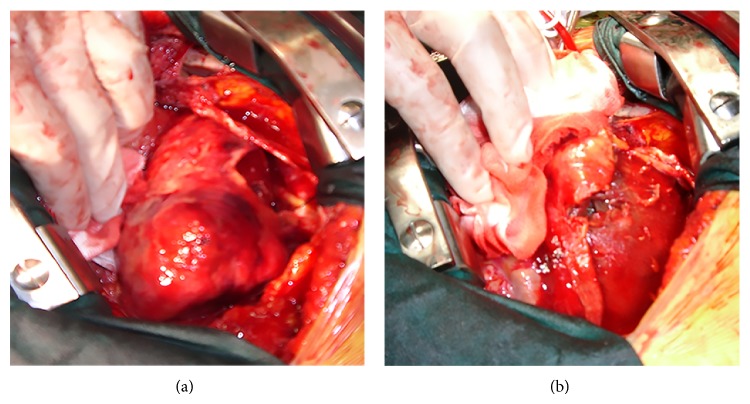
(a) Site of free wall rupture in the posterolateral wall surrounded by the intramyocardial dissecting plan (intraoperative view). (b) Intraoperative view of rupture side contained by myocardial adhesions.

A 61-year-old Caucasian female patient was admitted into the emergency room with complaints of progressive chest pain for 2 days, becoming continuous for the past 3-4 hours. The patient had a history of second time prosthetic aortic valve replacement and was under anticoagulation therapy. On physical examination, she presented a moderate general status and was hemodynamically stable. Electrocardiography (ECG) on admission showed sinus rhythm, ST segment elevation in leads D2, D3, and aVF, and ST segment depression in leads V1–3. Laboratory examinations revealed aninternational normalized ratio (INR) of 3.6 (normal, 0.8–1.2), creatine kinase muscle-brain fraction (CK-MB) level of 103 U/L (normal, 0–25 U/L), and a cardiac troponin I (cTnI) level of 6 ng/dL (normal, <0.01 ng/mL). She was diagnosed with an acute inferoposterior myocardial infarction (MI) and underwent emergency coronary angiography revealing spontaneous recanalization of the right coronary artery and also no significant stenosis in the left coronary artery. Medical treatment decision was taken and the patient transferred to the coronary care unit. Twenty-four hours after admission, she developed cardiogenic shock requiring intra-aortic balloonpump (IABP) placement. Physical examination revealed a new occurring systolic ejection murmur. Transthoracic echocardiography showed a left ventricular septum rupture (Figure 1); then, she was takenin for emergency surgery. During the operation, following adhesion's removal, a 2 × 3 cm^2^ rupture zone in the middle and the apical segment of posterolateral wall and a wide intramyocardial dissecting area around it extending into basal segments were detected (Figure 2) and the ventricular septum was intact. Intraventricular patch repair technic with autologous pericardial patch about 4 × 4 cm was used to exclude the ruptured area. A fibrin sealant (Tisseel, Baxter, USA) was applied between dissecting ventricular wall layers. Then, two strips of Teflon felt were applied at the borders of the ruptured area and were joined together by horizontal mattress sutures excluding the necrotic walls. Following the warming period, despite adequate hemostasis, hemorrhage around suture lines progressively increased, leading to the patient's death.

## 3. Discussion

Left ventricular free wall rupture (LVFWR) is the most common mechanical complication of acute MI occurring 6–8 times more often than rupture of the interventricular septum or papillary muscles [1]. Rupture into the pericardial sac usually results in sudden death unless adherent pericardium or scar tissue contains the rupture area leading to pseudoaneurysm formation [2, 3]. Only in very rare cases, the cardiac rupture might appear as a neocavitation entirely contained within the myocardial wall and forms an intramyocardial dissection (IMD); therefore, its diagnosis is usually challenging and in most cases it is observed postmortem [2].

In more than 90% of cases, rupture happens after the first heart attack and has a strong correlation with single-vessel disease reflecting lack of collateral circulation [4]. Risk factors for LVFWR include anterior wall infarct, large transmural infarction, age older than 60 years, preexistent hypertension, female gender, single-vessel disease [4], and absence of previous cardiac event [5]. Moreover, appearance of Q waves on the initial ECG and a peak CK-MB value higher than 150 IU/L seem to increase the risk of LVFWR [2, 5, 6]. All these factors indicate the lack of collateral circulation and clarify the reduced incidence of rupture associated with early reperfusion of infarct*-*related artery [4, 7].

The standard treatment of cardiac rupture is surgical repair containing the direct closure, infarctectomy, and endoventricular patch technics [8–11]. However, despite prompt diagnosis and treatment, mortality of acute LVFWR remains as high as 80% for some reasons [12]. Most patients have marked hemodynamic instability which was shown to be the most important predictor of outcome [13]. Moreover, necrotic tissue is generally too weak to hold sutures leading to uncontrollable bleeding due to suture lines tears. Therefore, the sutureless surgical techniques without cardiopulmonary bypass using glue and patch material have shown fairly good early and midterm results and gain popularity after the report of Padró et al. [14, 15]. Percutaneous intrapericardial fibrin-glue injection therapy (PIFIT) seems to be another promising therapeutic option for a patient with acute LVFWR with good midterm results [16]. Medical management seems to be alternative for very few patients remaining hemodynamically stable with medical and intra-aortic balloon pump counterpulsation supports [17].

Reported patient had a history of second time cardiac surgery causing excessive pericardial adhesions, which might contain the rupture and prevent the sudden death. However, an uncommon complication, IMD, appeared and might have deteriorated the patient's hemodynamic status despite maximal inotropic and IABP support. The prolonged INR level might have facilitated the development of intramyocardial dissecting hematoma. Moreover, prolonged INR level might have facilitated the development of intramyocardial dissecting hematoma.

## 4. Conclusion

Despite prompt diagnosis and promising treatment techniques, mortality of LVFWR still remains undesirably high, unless the adherent pericardium forms a ventricular pseudoaneurysm. Moreover, an intramyocardial dissecting hematoma might appear as a component of the rupture during the evolutionary process, in the very rare circumstance. These events have never been reported after MI, in a patient with a previous cardiac surgery.

## Supplementary Material

The video of modified apical four chamber view in transthoracic echocardiography showing the LV wall *defect and* color jet (A) *through it*

## Abbreviations

CK-MB: Creatine kinase muscle-brain fraction
cTnI: Cardiac troponin I
ECG: Electrocardiography
IABP: Intra-aortic balloon pump
INR: International normalized ratio
IMD: Intramyocardial dissection
LVFWR: Left ventricular free wall rupture
PIFIT: Percutaneous intrapericardial fibrin-glue injection therapy
MI: Myocardial infarction.
